# Factors impacting informed decision-making for patients with pancreatic cancer: a qualitative study

**DOI:** 10.1007/s00520-025-09878-9

**Published:** 2025-08-30

**Authors:** Patrick L. Quinn, Ann Scheck McAlearney, Jennifer Eramo, Sadie Chen, Jordan M. Cloyd, Laura J. Rush, Aslam Ejaz

**Affiliations:** 1 The Ohio State University Wexner Medical Center, Columbus, OH USA; 2 University of Illinois Chicago, Chicago, IL USA; 3 Department of Surgery, Division of Surgical Oncology, University of Illinois Chicago, Chicago, IL USA

**Keywords:** Pancreatic cancer, Informed decision-making, Qualitative study

## Abstract

**Purpose:**

Informed decision-making is an essential component of shared decision-making between patients and their cancer teams that promotes patient comprehension of their disease and available treatment options. The complex nature of multidisciplinary treatment for pancreatic cancer (PC) presents unique challenges for informed decision-making, for which barriers and facilitators are poorly understood.

**Methods:**

In this qualitative study, semi-structured interviews were conducted with PC patients seeking treatment at a Midwestern, high-volume, academic medical center to examine various aspects of informed decision-making, including patients’ understanding of treatment options, challenges in obtaining information, and how a decision aid may improve decision-making. Purposive sampling was used for recruitment and continued until thematic saturation was achieved. All interviews were audio-recorded, transcribed, and coded by the research team. Qualitative analysis was completed using an integrative approach involving both inductive and deductive methods.

**Results:**

Across 20 PC patients interviewed, the median age was 67 years, with 30% having metastatic disease (*n* = 6). Most patients reported having a knowledge gap about PC treatment options, requiring them to explore multiple resources to support their treatment decision-making. Furthermore, most interviewees noted that additional information in the form of a decision aid could be helpful in their decision-making process, particularly in understanding the advantages and disadvantages of treatment options, but also in introducing supportive care resources and improving their understanding of palliative care. However, concerns regarding the potential for providing too much information at once were common and perceived as potentially overwhelming.

**Conclusions:**

This qualitative study highlights the challenges that PC patients face in making informed treatment decisions and, therefore, in engaging in shared decision-making. Further research on patient-centered strategies, such as decision aids, is needed to improve the quality of both shared and informed decision-making.

**Supplementary Information:**

The online version contains supplementary material available at 10.1007/s00520-025-09878-9.

## Introduction

Patient-centeredness has been a key tenet of high-quality cancer care since its introduction in the 1960 s [1]. Though varying definitions exist, the basis of patient-centered care is a partnership between a patient and a clinician that incorporates the patient’s values, needs, and preferences into decision-making about healthcare choices [2]. Several vital elements of patient-centered care include patient individuality, communication, empowerment, and respect, with shared decision-making (SDM) between the patient and clinician recognized as the crux of patient-centered care [3, 4].

SDM refers to the collaboration between the patient and clinician to create treatment plans that align with a patient’s goals, an alignment that is also referred to as goal-concordant care [5]. For SDM to occur, patients and their caregivers must be well-informed regarding the nature of the disease, as well as about treatment options, including risks, benefits, and uncertainties (i.e., informed decision-making [IDM]) [6]. Particularly for patients with advanced cancer, prior studies have identified opportunities for improvements in SDM and IDM [7]. One such example is the development of care plans for patients with pancreatic cancer (PC). With a 5-year survival rate of only 13% and limited treatment options, as less than one-fifth of patients are eligible for curative-intent resection, a PC diagnosis poses obstacles to decision-making [8, 9]. PC thus requires complex multidisciplinary treatment, which can further complicate the SDM and IDM processes as more clinicians are included in care teams.

Previous studies have identified the lack of available treatment choices as a barrier to IDM among patients with PC [10, 11]. However, the specific barriers and facilitators to effective IDM, and therefore SDM, in patients with PC are not fully understood. Furthermore, the use of decision aids, tools designed to assist patients in understanding their treatment options and what matters most to them, has been underexplored within PC, with limited patient input on their value [12–14]. Therefore, the purpose of our qualitative study was to explore patients’ perspectives about various aspects of IDM, focusing on topics related to their information needs, their understanding of treatment options, their involvement in the decision-making process, and suggestions about how a decision aid may improve the process.

## Methods

### Study design and population

Patients with PC seeking treatment at a Midwestern, high-volume, academic medical center were recruited to participate in semi-structured interviews regarding their treatment experience. Inclusion criteria included English-speaking patients aged 18 years and older who had been diagnosed with exocrine pancreatic cancer at any point along their treatment course. Patients with precancerous tumors (e.g., intraductal papillary mucinous neoplasms), neuroendocrine tumors, or pancreatic metastases from another primary tumor were excluded due to differences in treatment regimens. Eligible participants were identified via prospective screening of both gastrointestinal oncology and surgical oncology ambulatory clinic electronic health records. Potential participants were then contacted by phone, at which point the study was explained. Verbal informed consent was obtained from patients who agreed to participate in the study. Interviews were conducted by phone at the patient’s convenience.

### Interview guide and process

A semi-structured interview guide was developed using a combination of evidence synthesis and expert opinion. The guide consisted of open-ended questions with follow-up probes based on patient responses. Questions in the interview guide asked about the decision-making process in selecting treatment options, perspectives on guideline-concordant care, and sources of information, and opportunities to address informational needs, specifically through the use of a decision aid (Supplemental Material). The interview guide was adjusted as needed after the initial interviews to ensure clarity of questions and improve the flow of the interviews. Interviews were conducted from March 2022 to May 2023 by three non-clinicians trained as interviewers and not involved with the patients’ care. Interviewees were given a gift card in appreciation for their participation. The study was approved by The Ohio State University Institutional Review Board (study ID 2020C0196) and followed the ethical standards set by the Declaration of Helsinki. All participants provided written informed consent before enrollment in the study.

### Data collection and analysis

All interviews were conducted by phone, audio recorded, and manually transcribed verbatim. Transcripts were reviewed and finalized by the research team before being uploaded to ATLAS.ti 23.1.1 (ATLAS.ti GmbH, Germany) for coding and analysis. An integrative approach, utilizing both inductive and deductive coding methodologies, was used for data extraction [15]. A preliminary codebook was created based on the questions asked in the interview guide and then iteratively refined after three independent coders coded the first three transcripts. After refining the preliminary codebook, four independent researchers coded the remaining transcripts until thematic saturation was achieved, indicating that no new themes emerged from the remaining transcripts. Discrepancies in coding were reviewed at regular team meetings and resolved through discussion. The final result was a thematic framework that synthesized participant perspectives into overarching themes, each comprising several subthemes and supported by illustrative quotes. This qualitative research process and manuscript development followed the Standards for Reporting Qualitative Research (SRQR) [16].

## Results

### Participant characteristics

Of 61 patients with pancreatic tumors identified from participating ambulatory clinics for possible enrollment, 20 patients were interviewed (Table 1). All participants identified as non-Hispanic White; the median age of interviewees was 67 years, with a majority of patients being female (*n* = 13, 65%). Less than a third of patients had metastatic disease (*n* = 6, 30%), and all but one patient were receiving systemic therapy (*n* = 19, 95%). A total of four patients (20%) were still employed at the time of the interview, with the majority of participants being currently married (*n* = 12, 60%).

**Table 1:** Patient information

Patient characteristic	*N* = 20
Age (years), median (IQR)	67 (63,73)
Sex, *n* (%)	
Male	7 (35)
Female	13 (65)
Cancer stage, *n* (%)	
I	3 (15)
II	9 (45)
III	2 (10)
IV	6 (30)
Treatment received^1^, *n* (%)	
Curative-intent resection	14 (70)
Systemic therapy	19 (95)
Radiation	7 (35)

^1^Not mutually exclusive

### Challenges for informed decision-making

Participants identified several challenges with IDM during their treatment, including sources of information, communication concerns, and being overwhelmed by information. These challenges are described in more detail below, with Table 2 presenting additional supporting quotations about reported challenges with IDM.

**Table 2:** Challenges in informed decision-making

Theme	Representative quote
*Concern with information sources*	“Once I was diagnosed, you kind of go onto Google, and you start looking around just for information. But my surgeon, he was like… ‘Don’t do that…a lot of that information is outdated. There’s been advancements made and whatever you’re reading in all likelihood has probably not caught up.’ Basically, you can read it but take it with a grain of salt.”
	“I really didn’t Google a whole lot because sometimes you end up sharing horror stories and so, I'm a little cautious with that. And…there was…a co-worker of a very good friend of mine whose husband had pancreatic cancer, and so she talked to me about her husband’s journey which was rather different than mine…and told me about the Whipple procedure and how difficult it had been for her husband, and honestly, scared me the living daylights out of me…”
*Rushed communication*	“Sometimes appointments can be rushed a little, but I know both [staff] and [doctor] are very good at listening to what I have to say…”
	“It always seems like they’re rushed when they come in. They sit down, but they’re wanting to move on…as they’re coming in…I’m sure because being the oncologist, that it’s a really harsh job…and I keep that in my mind all the time…They do listen when I speak, but I also felt like, as I was trying to answer questions with them that…they were speaking over me.”
*Use of jargon*	“Well, I think a layperson’s explanation of these two treatments I was offered would have been helpful. [Doctor]…he’s a scientist, he speaks from that frame of mind…it was pretty meaningless to me. So…to put these kinds of information into a layperson language, understandable language, but…with access to delving in more deeply should I want to or need to.”
	“He wrote out all these chemicals and this, that, and the other, and explained them. But it…was meaningless to me…I know nothing about these chemicals. You can explain to me…’ This does this, and this does that,’ but I’m…sitting there, so annoyed going, ‘So what? It doesn’t tell me what I should be doing.’”
*Information overload*	“I wanted [friend] to come so that in case I didn’t hear…because it’s a lot of information and also, to kind of be my backup…”
	“I got an awful lot of pamphlets and stuff that are printouts to take home and read. I had an awful lot of reading to do once I found out I had it…it is helpful, but it’s also confusing. The more you read the more you don’t know what you want…Sometimes it’s too much…”
*Need support with information processing*	“[Friend will] remember things that I need to ask, and…I always mean to write things down and, of course, we never do. So, she’ll kind of be my sounding board and the person who helps remember…what needs to be done, and what needs to happen.”
	“I didn’t know what I was doing, and I was glad my [child] was there, because I would not have remembered one word [doctor] said.”

#### Information sources

Participants reported that the defining features of their diagnosis and their treatment options were largely unknown. At the time of diagnosis, many participants proceeded with their own research, obtaining information from sources other than their medical team. The Internet was the predominant resource used, but family and friends were also consulted:

*My girlfriend was on top of everything, and if I had a question and there wasn’t a doctor around, I could ask her…she did a lot of research.*

While web sources can be a useful tool in helping patients learn about their disease and possible treatments, many patients recognize the added stress that personal research can create. As one participant explained:

*Sometimes you consume all that information from the internet, and you try and self-diagnose yourself, and you’ll drive yourself crazy. I kind of glanced through it, read what I wanted to read, and kind of let it go. I just have to trust in the man up above and the people that are taking care of me.*

Some participants acknowledged the anxiety that can result from online research and instead deferred information gathering to their friends and family.

#### Rushed communication

With a large knowledge gap among patients, participants described the constraints of time imposed by the medical system to obtain all the necessary information. During clinic visits, patients oftentimes felt that the medical team was in a hurry and would move on to their next task unless the patient interjected. As one patient suggested,

*It’s like they come in, do their thing, and then they’re gone…*

Several participants reflected that this feeling was strongest at the beginning of their treatment journey and diminished as their rapport with their medical team improved.

#### Use of jargon

Another concern regarding communication highlighted by participants was the use of medical or scientific terms. Many patients acknowledged feeling at a loss, particularly when discussing chemotherapy regimens because it’s a foreign topic to them:

*This whole thing was just so new. I mean, I have no perspective…I didn’t have any place where I could say…I’ve researched this and that and expect this…*

For some participants, making a medical decision was difficult because the complexity of the jargon and medical terms precluded a satisfactory understanding of their options.

#### Information overload

While patients were eager to learn more about their diagnosis and treatment options, too much information was often perceived as a burden instead of contributing to the decision-making process. This was impacted by the number and type of resources now available to patients. Patients leaving the clinic with a folder full of information can be overwhelmed by the amount of data they were expected to process and may have overlooked details that were important to them. In reference to treatment guidelines utilized by clinicians, a participant stated:

*Honestly, I don't remember anybody speaking to me about them, but I received a lot of information. I mean, I got booklets, I have fliers and tablets of information… it was overwhelming.*

#### Need support with information processing

The decision-making process occurs when patients are handling a new PC diagnosis, one well-known to be associated with poor outcomes and survival. Patients recognize that during these initial discussions, they are not at their sharpest and often will miss information presented to them or not recall the information at a later point. Several patients highlighted the importance of having a support network with them during clinic visits to capture the necessary information and be their spokesperson:

*If it weren’t for my [child], I wouldn’t have remembered half the things I was told. You just get overwhelmed…*

Beyond just emotional support, friends and family often serve as active participants in the clinical encounter, such as taking notes, asking clarifying questions, and ensuring that key information is understood and retained. Additionally, they may directly assist in the decision-making process for the patient, helping patients identify treatment options that align with their preferences:

*[My significant other] did more research than anybody I’ve ever seen. She had all kinds of information, and if she didn’t have it, the doctor had it. It was great to have her there to help me decide on what’s the best treatment.*

### Opportunities for informed decision-making

Participants were asked how they felt a decision aid would affect their decision-making and whether it was something that should be offered to patients. Participants identified several opportunities for improved IDM by utilizing a decision aid, including suggesting how the decision aid should be incorporated into the current treatment discussion and what content would be valuable. The following sections offer a more in-depth exploration of these opportunities, with Table 3 presenting additional relevant quotations about the highlighted opportunities.

**Table 3:** Opportunities to improve informed decision-making

Theme	Representative quote
**Decision aids**	
*Reference document*	“To have everything…all laid out in front of you so you could see it. It helps you to understand it way easier…I totally think that would be a major bonus.”
	“I don’t know what I don’t know. I’ve never had pancreatic cancer before, so I didn’t know in the very beginning, in particular, a lot of the things that were, or be able to reference back to it. So, if there was documentation like you’re talking about or a decision-making tree, I think it makes people think a little bit more about their treatment options and the decisions that they have to make. Because I do think that sometimes you’re only half listening.”
*Patient aid*	“An aid probably could help clarify, or at least give me somebody to kind of act like my friend, someone that could be like an advocate. I know you guys have advocates here, but someone…who can say…’ Let’s talk about some of the points that the doctor discussed and how do you feel about that.’
	“That appointment…with [doctor]…if I had…help, like, not being rushed to make a decision…had somebody to talk to over the weekend about this…a patient aid of some sort…that would have been helpful.”
**Content**	
*More information is better*	“No matter how you have to come about getting it…you want all the information as best as you can get.”
	“I’m definitely in the category of the more information, the better…”
*What questions to ask*	“I think so (a decision aid would have helped to better communicate). I could have asked some more intelligent questions…maybe some of them I could have found out on my own, but, probably, I could’ve asked something that would have been…easier for him to understand the question.”
	“I didn’t know what to ask…I was just dumbfounded that they found anything, and…whatever they suggested, I took…”
*Supportive care*	“When I was first diagnosed, just maybe understanding and discussing Palliative Care [in a decision aid] …I think it’s important…I think it would maybe help…Help you make decisions on further care.”
	“I didn’t know what to expect. I was already scared. You need to go ahead and let me know that that’s one of my choices for help, and…I’m definitely going to go, ‘Anything you can give me for making my quality of life better now I’ll take it.’ So, I think it needs to be…maybe the very first visit you have with that doctor

#### Decision aid—reference document

While generally receptive to the idea of a decision aid, interviewees differed in the ways they described how such a tool could help. For instance, some patients stated that having a decision aid that acted as a reference document could be useful, particularly in helping them remember key points regarding their treatment options:

*In the beginning, you’re still in shock…You’re introduced to this whirlwind of appointments, and this is how this is going to work, and here’s what we're going to be doing. You don’t necessarily hear everything. But if they are given something to review after time, I think that they would go back to their documentation and review it.*

This connects back to those patients who had a sense of information overload during the first consultation, often feeling that they could not remember important details regarding their diagnosis or treatment plan.

### Decision aid—patient advocate

For some interviewees, the ideal decision aid they described was not a physical document, but rather a person within the healthcare system who could discuss treatment options with them in further detail. As one patient explained:

*I think walking me through options, giving me more information…instead of giving me a pamphlet full of papers. You’re better at listening to somebody than trying to consume it by reading it…if I had somebody that walked me through it step by step…I don't think I would have changed anything that I've done, but I just think it would have been a little bit more understanding.*

Participants were not always looking for additional information in the form of a decision aid but rather wanted the content already presented to them reinforced in a way that was easily comprehensible and could help guide them through their treatment journey. Patients acknowledged that clinicians are not always available to answer their questions, therefore the expectation was that a patient advocate would not necessarily be a clinician but another member of the care team. For some participants, they admitted that their comprehension was much more dependent on discussions rather than reading or watching other information resources.

#### Content—more information is better

For some participants, any additional information was considered beneficial, regardless of the format in which the information was delivered. These interviewees explained that they were looking for any meaningful tool that could support them with their treatment decisions. As voiced by one participant:

*I think every bit of assistance is going to be helpful to me…. We want to be informed, and if there was one more way of being able to be informed, one more way to communicate, that it’s going to make our lives easier.*

In conjunction with the previous section on information overload, this highlights that there is a spectrum of information needs, with some patients wanting the minimum amount of information needed to make decisions and others interested in having all available information.

#### Content—what questions to ask

Participants reportedly wanted time to deliberate over their available options and not feel rushed to come to a decision. However, these interviewees also conveyed trust in their care team and noted feeling that they were receiving the best medical care with their best intentions in mind, regardless of whether they were deferring decision-making to the clinician. As one patient put it:

*I didn’t feel compelled to question what they were expressing to me because… I had this feeling of trust about it all…*

This is not to say that participants were not interested in being involved in their care, as several participants expressed interest in their treatment plans, but many interviewees reflected that they were not well-equipped for these discussions. Participants noted that they often did not know what questions would be relevant or important in deciding a treatment plan:

*At the beginning, I was so overwhelmed that there were a lot of things I didn’t think to ask…*

For some participants, a decision aid reportedly might have been useful prior to their clinic appointments as a way to help guide them through the discussion.

#### Content—supportive care resources

One area of content that participants thought would be useful in a decision aid was supportive care resources. For some patients, just being given the information can make all the difference:

*I got a flyer that had all kinds of different things that were available and that kind of stuff…it really made a difference to me when the music therapist and the art therapist came and saw me and talked about what’s available and how they can help.*

One aspect of supportive care that would benefit from further explanation is palliative care, as many patients acknowledged being confused by the term or associating palliative care with only end-of-life or hospice care. Some patients were altogether unfamiliar with the term, as highlighted by one interviewee who stated:

*I’m not even sure what the word [palliative care] means…*

One avenue to invite discussion regarding palliative care would be through a decision aid. Some patients suggested that discussing palliative care earlier could be beneficial for future patients,

*The more information (about Palliative Care) a patient has, the better. And I think if you give people in that position, let them know what their options are, here's the choices…I think that would be helpful*

Correspondingly, for patients who are not ready or are uninterested in early discussions regarding supportive care, a decision aid could act as a reference document, available when the patient becomes more receptive to receiving the information.

## Discussion

The crux of patient-centered care is having patients be actively involved in the decision-making process, allowing them to co-design treatment plans with their goals and priorities in mind [4]. For patients to effectively participate in their medical decision-making, they must be well-informed about their disease process and its treatment options [5]. Pancreatic cancer poses a challenge to IDM due to limited patient awareness, poor prognosis, and complex treatment options [7]. Within our qualitative study, we explored challenges to effective IDM with PC patients and investigated how a decision aid might remedy such issues. We found that while the content provided to patients was not necessarily lacking, the methods by which the information was delivered and received by patients created issues. Participants were open to the use of a formal decision aid and/or introduction of a patient advocate, and reported that they thought it would be a good opportunity to introduce supportive care options to patients.

One of the challenges identified by our study participants was the timing of information delivery. While patients are dealing with the shock of a pancreatic cancer diagnosis, they feel overwhelmed with the responsibility to absorb all the necessary information to make a treatment decision after a short clinic visit. Emotions influence how patients handle information, with many patients citing that they had forgotten or had not heard crucial information relevant to their disease process. A similar theme was found by Geessink et al. who interviewed older PC and colorectal cancer patients and found that remembering the information provided was difficult [17]. Previous interviews with patients with hepatopancreatobiliary cancers also found that the short time interval to receive and process complex treatment options limited the ability to make an informed decision [18]. Furthermore, the expected quick turnaround can place added pressure on patients, resulting in the deferral of treatment decision-making to others [10].

An additional challenge is understanding how much information patients are interested in receiving and titrating discussions appropriately for each patient based on individual information preferences. As our study highlights, there is a spectrum of information needs among patients, with some patients wanting as much information as possible, and others wanting just enough to make treatment decisions. One proposed solution is to incorporate the Ask-Tell-Ask framework into treatment discussions [19]. This is a process in which a provider first explores the patient’s baseline knowledge and information needs, then provides information based on the initial assessment, and then follows up with the patient to identify if any information gaps remain [20]. An alternative solution that can be employed before the clinical encounter is pre-visit preference assessments. These are short surveys that can help elicit a patient’s treatment goals before discussion with the medical team, allowing providers to tailor discussions based on a patient’s responses. Within prostate cancer, the implementation of a pre-visit preference assessment was associated with improved satisfaction with care and treatment decisions and reduced decisional regret [21]. Such processes have not been directly examined within pancreatic cancer and warrant further investigation.

Providing highly complex information in a limited period of time proves to be a challenge for cancer clinicians in the current healthcare landscape. Clinicians face high-volume appointment schedules and often do not have the liberty to extend the duration of patient appointments without impinging on other patients’ time. Various solutions that have been proposed include developing a decision-making timeline with the patient, spreading decisions over multiple consultations, and using time between consultations more effectively [22]. A tool that has been implemented as a way to better facilitate decision-making conversations in oncology is a decision aid [12]. In our study, patients were open to the idea of introducing a decision aid to the process, but they diverged in how they imagined the tool would work. In practice, the use of decision aids among patients with PC is limited and shows mixed results with respect to its impact on improving SDM [13, 14]. Rather than a traditional decision aid, patients in our study suggested that something to prompt discussion and help them elicit important information from their physician would be helpful. One decision aid designed for that purpose is a question prompt list (QPL), a list of commonly asked questions provided to patients at the time of consultation. In a pilot trial, Oba et al. found the combination of patient coaching and QPL was useful for older PC patients [23]. Additionally, the Pancreatic Cancer Action Network (PANCAN) provides a QPL on its website, but its uptake and usefulness in clinical discussions for PC patients have not been evaluated [24]. With many patients using online resources for information, an embedded QPL within the clinic could be valuable as a result of the limited quality and readability of internet sources [25, 26]. Given the lack of studies evaluating the use of decision aids, further research is needed to investigate the benefits and challenges of various methods to support PC patients’ informational needs.

Patients in our study also noted that a patient advocate could be helpful to them in the decision-making process, consistent with findings from prior research [27, 28]. While patient navigators have been introduced as a way of improving access to care and assisting patients with the logistics of cancer care, patients often look to them as patient advocates for support regarding their cancer treatment [29]. Similarly, previous studies have reported that patients may look to their oncology nurses as a type of patient advocate for assistance with treatment decisions [30].

A final area that was identified by study participants that needed further explanation was palliative care, as many reportedly did not understand the intention of palliative care, thinking that it was solely hospice care. This misperception has been previously noted among cancer patients, with patients often interpreting discussions surrounding palliative care as a sign of hopelessness [31]. Studies evaluating the implementation of early palliative care in advanced cancer, however, have found improvements in patient quality of life and symptom intensity, suggesting this may be an opportunity for PC patients [32]. Patients in our study noted that a decision aid might be an effective way to introduce supportive care and patient treatment options, leaving patients to decide on their own whether it might be something they would like to pursue. This finding is similar to results reported by Tseng et al. who found that the implementation of an SDM model in addition to a decision aid increased the acceptance of palliative care for patients with advanced PC [13]. Further research evaluating the impact that decision aids can have on PC patients’ perceptions of palliative care can help inform this approach to improve SDM in the future.

### Limitations

There are limitations to our study that must be considered. First, our research involved patients seeking care at a single institution. Additionally, given the small sample size, how different patient factors, such as age or stage at diagnosis, might influence patient perspectives was not able to be explored. Furthermore, our cohort only consisted of non-Hispanic White patients, meaning that our findings may not represent the patient experience of those from minority groups. Nonetheless, our study included patients across all stages of PC, enabling us to comprehensively evaluate the different needs of PC patients throughout their treatment journey. Finally, our study focused only on patients’ perspectives of the decision-making process. However, these results may provide useful information for cancer providers seeking to improve their IDM process.

## Conclusions

It is essential for cancer clinicians to recognize the difficulties patients face when processing new information and making treatment decisions. Our qualitative study reiterated common challenges found in IDM for advanced cancer patients, while adding new patient perspectives regarding aids used to assist patient decision-making. Future studies examining how adjunct processes, such as decision aids, can improve the shared and informed decision-making process will be important to increase decision-making and the patient-centeredness of care for pancreatic cancer patients.

## Supplementary Information

Below is the link to the electronic supplementary material.

Supplementary file1 (DOCX 37 KB)

## Data Availability

No datasets were generated or analysed during the current study.
