# Supplementary material for: Factors impacting informed decision-making for patients with pancreatic cancer: a qualitative study
Source: Support Care Cancer. 2025 Aug 30;33(9):825. doi: 10.1007/s00520-025-09878-9 (PMC12398429; doi:10.1007/s00520-025-09878-9)
Supplement: Supplementary file 1 — Supplementary file1 (DOCX 37 KB) [file 520_2025_9878_MOESM1_ESM.docx]

**Patient Interview Guide**

**INTRODUCTION**

Treatment for pancreatic cancer is complex and involves care from numerous specialized providers. The purpose of our research is to understand how patients with pancreatic cancer make decisions about their treatment goals and whether they choose to receive palliative care. The main goal of our study is to improve the support that patients receive in making these decisions from the onset of their diagnosis. I will now take you through an informed consent process.

*After consent has been read, state:*

“I would like to make a recording of our discussion, so that I can have an accurate record of the information that you provide to me. The recording and transcripts will be kept confidential, using a unique study code and secured in password-protected files, only accessible by researchers with this study.”

“Do you have any questions about this research?

Do you agree to participate, with this study?

If so, let’s begin….”

**OVERVIEW OF INTERVIEW TOPICS**

In this interview, I will ask you a series of open-ended questions to get your perspectives about several topics related to your pancreatic cancer care. These topics include:

- Section 1: Background
- Section 2: Decision-making process in selecting treatment options
- Section 3: Values and preferences for guideline-concordant care vs. goal-concordant care
- Section 4: Opportunities to address your informational needs
- Section 5: Perceptions of palliative care and opportunities to address your needs

INTERVIEW

**Section 1: Background**

- To start, for our records, when were you diagnosed with pancreatic cancer?
- Are you currently receiving treatment for your pancreatic cancer?
  - If yes, what types of treatment are you getting (e.g., chemotherapy, radiation, surgery, or palliative care)?
    - Where do you go to get your treatment(s)?

**Section 2: The Decision-Making Process for Selecting Treatment Options**

(Note to Interviewer: This set of questions is designed to elicit information regarding how well-aligned the patient’s decision-making process was with the first two steps in the SHARE Approach: Step 1) Seek your patient’s participation and Step 2) Help your patient explore and compare treatment options.)

The purpose of the next set of questions is to learn about how you made treatment decisions.

- Did your doctor seek your participation in the treatment decision making process?
- Did your doctor help you to explore and compare different treatment options for pancreatic cancer?
  - If yes, please describe the options that were presented to you.
  - How did your doctor help you explore and compare these options?
    - Probes: Referral to a nurse educator, pharmacist, palliative care clinician, other type of clinician; website; pamphlets; national treatment guidelines/recommendations; other resources
      - - *If patient received help*, which resources were most helpful? What else might have been helpful?
        - *If patient did not receive help*, what might have been helpful?
- Did you get information regarding treatment options for pancreatic cancer from sources other than your doctor?
  - Prompt: Websites? Friends/family? Support group sites?
  - *If yes*, did you share this information with your doctor? How was it received?
- What was most important to you when making your decision?
  - Prompt: Life goals/priorities, doctor recommendation
- What questions did you ask your doctor about the treatment options for pancreatic cancer?
  - Prompt: Pros/Cons? Potential risks/side effects? Overall prognosis?
- What was missing in the discussion?
  - Prompt: Was there information that you wanted, but did not receive?
  - Prompt: Did you feel well informed about the different treatment options?
- Did you feel you had an option to choose (or choose not) to receive treatment for your pancreatic cancer?
  - Prompt: How much of a “say” did you personally have when making your treatment decision (versus your physician? Family members?)
- How did you ultimately come to your decision?

**Section 3: Patient Values and Preferences for Guideline-Concordant Care vs. Goal-Concordant Care**

Note to Interviewer: This set of questions is designed to elicit information regarding how well-aligned the patient’s decision-making process was with the third step in the SHARE Approach: Step 3) Assess your patient’s values and preferences).

Several evidenced-based guidelines set the standard of care for how to treat pancreatic cancer. Doctors refer to these guidelines when recommending treatment options to their patients. In addition to evidence-based guidelines, doctors refer to the recommendations of agencies like the National Cancer Institute’s Division of Cancer Control and Population Sciences regarding best practices for patient-provider communication.

- Were you made aware of any of these national treatment guidelines or recommendations when first diagnosed?
  - At any time since your diagnosis?
- Do you believe you are receiving the best available treatment according to these evidenced-based guidelines?
  - Why/why not?
- Do you believe you are receiving the best available treatment according to your personal priorities?
  - Why/why not?
- Is there anything about your cancer treatment that you wish could have been/could be done differently? (Please explain.)
- Out of all your doctors that you see for pancreatic cancer, which ones do you feel more comfortable opening up to and talking about your cancer treatment?
  - Why?

**Section 4: Opportunities to Address Patients Informational Needs**

(Note to Interviewer: This set of questions is designed to elicit information regarding how a decision aid tool might help with the fourth step in the SHARE Approach: Step 4) Reach a Decision with your Patient)

The goal of our study is to improve the treatment decision making process for people with PC. The use of decision aids has been proven to improve shared provider-patient shared decision-making in patients with other types of cancer (not PC). A patient decision aid is a tool that provides patients with information about their treatment options and potential outcomes. The goal of our study is to create a decision aid to help patients with PC to clarify and align their medical care with their personal values.

- Looking back on your decision-making experience, how could a decision aid have helped you make a decision that was aligned with treatment recommendations and your personal values?
  - Prompts: Provide existing examples of how decision aids are used in other cancer types
- Do you think a decision aid would have helped you better communicate with your doctor about your treatment decisions?
  - Prompt: Consider the conversations you had with your doctor, the resources you found helpful, and how a decision aid could have helped with the process

**Section 5: Patient Perceptions of Palliative Care and Opportunities to Address Patient Needs**

(Note to Interviewer: This set of questions is designed to elicit information regarding how a decision aid tool might help with the fourth step in the SHARE Approach: Step 4) Reach a Decision with your Patient)

In addition to treatment options such as surgery and chemotherapy, there is also an option for palliative care.

- Have you heard of palliative care?
  - *If yes,* What do you think palliative care entails?
    - Prompt: How does hearing the term “palliative care” make you feel?
  - *If no,* provide definition of palliative care:

Palliative care supports patients and their families in an approach that is focuses on the patient and aims to optimize the patients’ quality of life. Palliative care promotes **goal-concordant care** by exploring patient and family goals and informing decision making when patients, their families, and their providers are weighing the potential risks and benefits of various treatments.

- Now that you have heard this description of palliative care, do you think it could help you with addressing your cancer treatment needs?
  - *If yes,* how so*?*
  - *If no,* why not?

How do you suggest we should incorporate palliative care into a decision aid?

**Wrap-up/Conclusion**

- Is there anything else you would like to tell us about your cancer care experience or how you think a decision aid might be useful?

**Thank you** for your time and participation! Your comments are extremely helpful to our work.
